# Transcriptomes reveal expression of hemoglobins throughout insects and other Hexapoda

**DOI:** 10.1371/journal.pone.0234272

**Published:** 2020-06-05

**Authors:** Hollister W. Herhold, Steven R. Davis, David A. Grimaldi

**Affiliations:** Division of Invertebrate Zoology, American Museum of Natural History, New York, New York, United States of America; Universite de Lausanne Faculte de biologie et medecine, SWITZERLAND

## Abstract

Insects have long been thought to largely not require hemoglobins, with some notable exceptions like the red hemolymph of chironomid larvae. The tubular, branching network of tracheae in hexapods is traditionally considered sufficient for their respiration. Where hemoglobins do occur sporadically in plants and animals, they are believed to be either convergent, or because they are ancient in origin and their expression is lost in many clades. Our comprehensive analysis of 845 Hexapod transcriptomes, totaling over 38 Gbases, revealed the expression of hemoglobins in all 32 orders of hexapods, including the 29 recognized orders of insects. Discovery and identification of 1333 putative hemoglobins were achieved with target-gene BLAST searches of the NCBI TSA database, verifying functional residues, secondary- and tertiary-structure predictions, and localization predictions based on machine learning. While the majority of these hemoglobins are intracellular, extracellular ones were recovered in 38 species. Gene trees were constructed via multiple-sequence alignments and phylogenetic analyses. These indicate duplication events within insects and a monophyletic grouping of hemoglobins outside other globin clades, for which we propose the term *insectahemoglobins*. These hemoglobins are phylogenetically adjacent and appear structurally convergent with the clade of chordate myoglobins, cytoglobins, and hemoglobins. Their derivation and co-option from early neuroglobins may explain the widespread nature of hemoglobins in various kingdoms and phyla. These results will guide future work involving genome comparisons to transcriptome results, experimental investigations of gene expression, cell and tissue localization, and gas binding properties, all of which are needed to further illuminate the complex respiratory adaptations in insects.

## Introduction

The colonization of land by arthropods in the Paleozoic required profound changes in respiration. These animals transitioned from a system of gills and respiratory proteins, like hemocyanins, to breathing air. Hemoglobins are also well known for their common role in respiration, although they have various other roles too, discussed below. Hemoglobins are globular proteins of 140–150 aa length, usually comprised of eight 3-over-3 α-helical segments (A-H), with Fe^++^ that binds O_2_, CO, NO, and CO_2_. The amino acid sequences can vary widely among taxa, but to preserve the oxygen-binding properties there are two highly recognizable regions in hemoglobin, comprising the characteristic "globin fold": Phe and His occur at positions CD1 and F8, respectively, and hydrophobic residues occur in each of the α-helical segments [1]. Hemoglobins occur in at least five kingdoms of life and 12 phyla of animals from protists to vertebrates [1], but despite their apparent antiquity their expression among animals is presently known to be sporadic, and absent in most orders and species of the largest radiation, the insects [1,2].

Hemoglobin expression has been shown to occur in approximately 19 species of insects belonging to 14 families and five orders of insects (Table 1). Additional species have been identified as possessing hemoglobins, using sequence identity (e.g., [3,4]) genome and transcriptome annotations (e.g., [4] and other methods (e.g., [5]). These are not included in Table 1, only hemoglobins that have been functionally, experimentally, or biochemically characterized are listed. Some have been known for many years, particularly in *Chironomus* midge larvae [6–8]; stomach bot flies, *Gasterophilus* [9]; and the backswimmer bugs *Anisops* and *Buenoa* [10,11].

**Table 1:** Insects with characterized hemoglobins.

ORDER Genus—Species	Family	Life Stage	Forms	Respiratory?	Location	Fe2+ binding/Structure	Refs.
**DIPTERA:**							
*Chironomus* spp.	Chironomidae	larvae	various	Yes	extracellular	Monomers, dimers	[12–16]
*Chironomus tentans*	Chironomidae	adult	Ctglob1	no?	extracellular		[16–19]
*Drosophila melanogaster*	Drosophilidae	adult	Dmglob1	probably*	intracellular	Hexa-coordinate/ Monomer	[4,14–16,20,21]
			Dmglob2	no?	intracellular	Monomer	[4,14–16,20,21]
			Dmglob3	no?	intracellular	Monomer	[4,14–16,20,21]
*Anopheles gambiae*	Culicidae	larva	Agglob1	probably*	intracellular		[15]
			Agglob2	probably*	intracellular		[15]
*Aedes aegypti*	Culicidae	larva	Aaglob1	probably*	intracellular		[15]
			Aaglob2	probably*	intracellular		[15]
*Gasterophilus intestinalis*	Oestridae	larva	Giglob1	Yes	intracellular	Pentacoordinate/ Dimer	[4,9,16,22,23]
*Glossina morsitans*	Glossinidae		Gmglob1	probably*	intracellular		[4]
**HYMENOPTERA**:							
*Apis mellifera*	Apidae	all?	Amglob1	probably*	intracellular		[4][16]
**HEMIPTERA**:							
*Acyrthosiphon pisum*	Aphididae	all?	Apglob1	probably*	intracellular		[4]
*Aphis gossypii*	Aphididae	all?	Agglob1	probably*	intracellular		[4]
*Anisops assimilis*	Notonectidae	all?	Aaglob1	Yes	intracellular	Penta-coordinate/ Monomer, hexamer	[3,16]
*Anisops deanei*	Notonectidae	all?	Adglob1	Yes	intracellular	Penta-coordinate/ Monomer, hexamer	[3]
			Adglob2	Yes	intracellular	Penta-coordinate/ Monomer, hexamer	[3]
			Adglob3	Yes	intracellular	Penta-coordinate/ Monomer, hexamer	[3]
*Buenoa confusa*	Notonectidae	all?	Bcglob1	Yes	intracellular	Monomer, dimer	[16,24,25]
*Buenoa macrotibialis*	Notonectidae	all?	Bmglob1	probably*	intracellular	Monomer, dimer	[3]
*Macrocoryxia geoffroyi*	Coryxidae	all?	Mgglob1	Yes	intracellular		[16]
*Nilaparvata lugens*	Delphacidae	all?	Nlglob1	probably*	intracellular		[3]
**COLEOPTERA**:							
*Dascillus cervinus*	Dascillidae	all?	Dcglob1	probably*	intracellular		[4,16]
*Tribolium castaneum*	Tenebrionidae	all?	Tcglob1	probably*	intracellular		[4,16]
**LEPIDOPTERA**:							
*Bombyx mori*	Bombycidae	all?	Bmglob1	probably*	intracellular		[4,16,26]
*Samia cynthia ricini*	Saturniidae	all?	Scglob1	probably*	intracellular		[26]

*Inferred on basis of Hb-producing cells located near or in tracheal cells, or gene identity.

Extensive studies by Burmester and colleagues have revealed the surprising occurrence of hemoglobins in insects that seem to have little or no oxygen limitations, including the well-known *Apis mellifera*, *Drosophila melanogaster*, *Anopheles* mosquitoes, and a dozen species of true bugs, beetles, and moths (Table 1). Most of these characterized hemoglobins are implicated in respiration and associated with the tracheal system, though a few are involved in other physiological and developmental processes (e.g., [1,27,28]). The majority appear to be localized intracellularly, likely cytoplasmic or in the cell membranes of tracheocytes and adipocytes; a few are extracellular, dissolved in the hemolymph and are responsible for its red coloration. Although relatively few insect hemoglobins have been studied in detail, their copy number is variable and appear to show stage- and tissue-specific expression differences [27]. Substantial hemoglobin structural variation also occurs in insects, ranging from monomeric to di-, tetra-, and hexameric quaternary structures. Binding schemes of the heme Fe^++^ in the deoxygenated state varies from penta- to hexacoordinate, the functional implications of which remain unclear.

There are several reasons why, until now, hemoglobins in insects have been thought to occur so sporadically.

**1.** *Assumptions among most biologists that hemoglobins are entirely respiratory in function*. Although O_2_ transport may be the most common function of Hbs, some forms have varied functions, particularly in invertebrates: in the regulation and detoxification of NO [29], acid-base regulation, oxidase-peroxidase activities, and reactions with sulfide and its transport [1]. Other functions relate to O_2_ metabolism, such as sensing [30], facilitating O_2_ diffusion, and O_2_ scavenging [1]. Some hemoglobins, in fact, apparently serve to protect cells against reactive oxygen species (ROS), similar to vertebrate myoglobin. Discontinuous breathing in insects has been shown to be an adaptive response to ROS [31].

**2**. *Hemocyanins are the primary respiratory proteins in Pancrustacea (Crustacea including insects)* [2]. Hemocyanins are Cu^+^ containing proteins found in some mollusks, Crustacea (including basal insects), and the sister phylum to arthropods, the Onychophora (velvet worms)[2]. They are, so far as known, always freely dissolved in hemolymph, and may be derived from phenoloxidases [2]. They appear to be lost in a clade of Crustacea (Branchiopoda + Copepoda + Thecostraca, where they are replaced by hemoglobin), in the paleopterous insects (Ephemeroptera and Odonata), and in the holometabolans + paraneopterans [2]. Expression of hemocyanin in polyneopteran insects (stoneflies, roaches, mantises, orthopterans, earwigs, etc.) appears relegated to embryos, so these proteins are thought to be involved either in development or in respiration of the insect embryo among basal lineages of insects.

**3.** *The adequacy and efficiency of arthropod respiration via tracheae and book lungs*. Every terrestrial group of arthropods has a complex system of invaginations into the body for the direct delivery of air to tissues, principally in the form of tracheae for insects, myriapods (centipedes and millipedes), and some arachnids (e.g., solifugae [32]), or as book lungs in many spiders and all scorpions [33], and pleopod lungs in pill bugs (Oniscoidae) [34]. Because of ambiguous relationships among the 11 orders of arachnids [35], it is difficult to determine exactly how many times arachnids independently evolved tracheae, but based on structure it may have been five times (e.g., [36–38]). The implication of this remarkable, repeated convergence is that tracheae and book lungs are not only necessary for arthropod breathing on land, they are entirely adequate. Indeed, our study was initiated as a result of our work on insect tracheae. Despite great adaptive variation in the tracheal system among hexapods, we sought to determine if there might be any physiological compensation for differences in respiration.

**4.** *Insect Hb expression depends upon exceptional life histories where there is strong selection for oxygen-absorption*. Hb imparts the deep red color to certain *Chironomus* larvae that live in hypoxic habitats like the sediments of eutrophic and polluted non-saline water. *Chironomus* has been intensively studied also because the larval hemoglobins are uniquely dissolved in hemolymph rather than within cells, and multiple forms occur in some species (larval *C*. *tentans*, for example, has up to 40 forms of hemoglobin) [16,39]. Larvae of the horse bot flies, *Gasterophilus*, are embedded into the host stomach wall, obtaining oxygen through air that the horse ingests [9,22]. *Anisops* and some other Corixidae are active predators in ponds, which breathe from bubbles trapped against the body (a plastron) during dives. Their Hb is a source of oxygen later in the dive, so that the plastron is not depleted and the bug can retain buoyancy [11,40]. Although it is not an insect, it merits mention that the only copepod that is known to have hemoglobins, *Benthoxynus spiculifer*, lives in the hypoxic water of deep-sea thermal vents [41].

Here we show through transcriptomics that hemoglobin expression is in fact ubiquitous among insects, including in their hexapod relatives. Based on transcript sequence identity we are able to speculate on the possible function(s) of some, but hemoglobin functions in most of the species will require experimental study. Our results are a rigorous test and confirmation of Burmester's [2](pg. 797) statement that " …; apparently many—but probably not all—pancrustacean genomes or transcriptomes harbor an HbL [hemoglobin-like] gene", and the more recent hypothesis [27](pg. 230) that “… a glob1-like gene belongs to the standard repertoire of insects …”. The results have implications for our understanding of globin genes, which are primary models for the evolution of gene duplication and molecular function (e.g., [42]).

## Results and discussion

### Searching for novel hemoglobin sequences

Previous studies have used bioinformatic methods to investigate the presence of hemoglobins in certain plants, bacteria, and eukaryotes, having focused on individual specimens in order to characterize and determine the functions of single hemoglobins [11,22,43]. The objective of our study was an extensive, comparative approach to test if hemoglobins are present in the genome, but more importantly, if they are expressed across all orders of Hexapoda. Using standard NCBI Transcriptome Shotgun Assembly (TSA) Database online search tools, 845 hexapod transcriptomes, totaling over 38.2 Gbases, were retrieved in June 2018 [44]. In addition to representatives from all 29 recognized insect orders, one transcriptome each from Remipedia and Protura, two from Diplura, and 8 from Collembola were included (See S1 File for identifiers and accession information). Remipedia is a close crustacean relative of hexapods; the latter 3 comprise the Entognatha, which is the living sister group to the Insecta. As 78 taxa had multiple transcriptomes in the database, the final dataset contained a total of 716 species. Species with duplicate transcriptomes were included because gene expression can be dependent on factors such as development stage or tissue type and the aim of the study was to investigate the overall presence of hemoglobins in hexapods.

**Fig 1:**
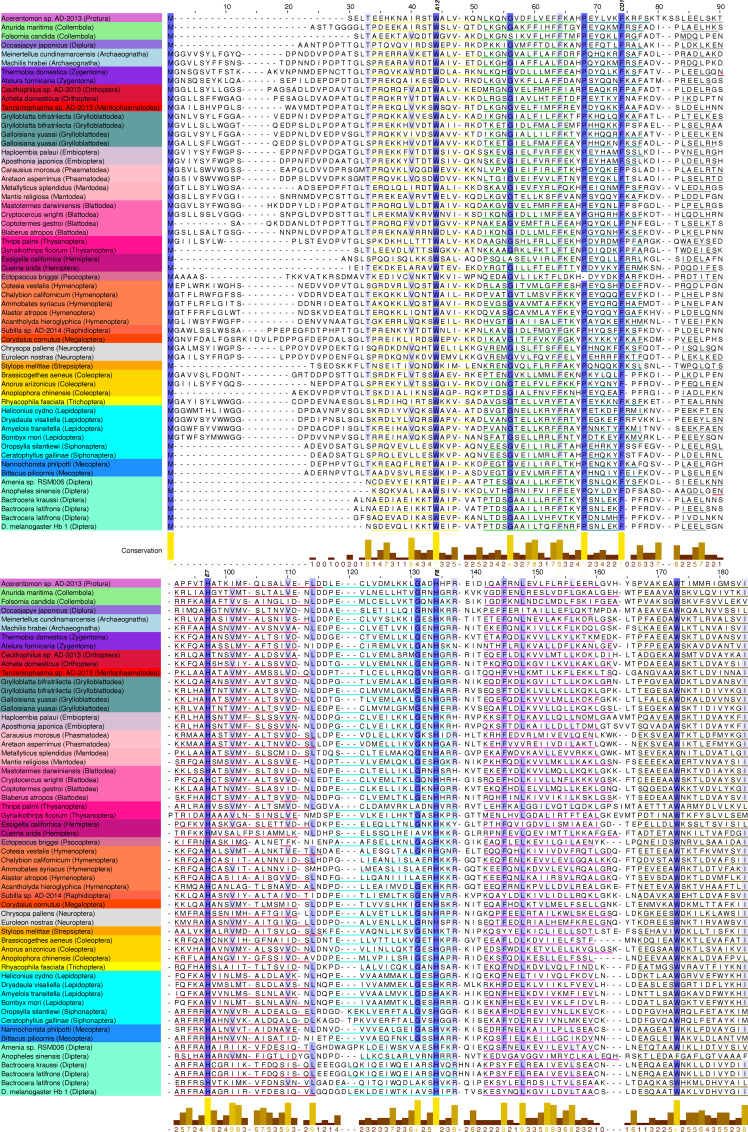
Hemoglobin exemplar MSA. Multiple sequence alignment of hemoglobin amino acid sequences selected from 60 exemplar species across Hexapoda, truncated at 180 residues (end of helix H) to conserve space. Background colors correspond to specimen orders (Diptera, etc) for all subsequent figures. Secondary structure predictions are annotated using colored underlines to indicate α-helices corresponding to helices detailed in S3 File. Helix colors are as follows: A = yellow, B = light green, C = dark green, D = gray, E = red, F = teal, G = magenta, and H = brown. Locations of functionally important residues (in particular, tryptophan at A12, phenylalanine at CD1, and histidine at E7 and F8) are noted as blue vertical bars. Conservation annotations computed by JalView, measuring the number of physio-chemical properties conserved [45].

Potential hemoglobin amino acid sequences were uncovered with a two-tiered BLAST search using insect hemoglobin amino acid sequences from the literature as target genes (S1 Table). Sequences were identified as hemoglobins via multiple sequence alignments to confirm functionally important residues, secondary- and tertiary-structure predictions and alignments to verify the characteristic 3-over-3 α-helix arrangement, and cellular localization predictions to determine probable functionality (see Methods and Materials for overview, and S2 File for full implementation details on bioinformatic workflow). Fig 1 presents a 60-sequence exemplar subset of identified hemoglobins with representatives from all 32 hexapod orders to illustrate alignment of functionally important residues and secondary structure predictions.

**Fig 2:**
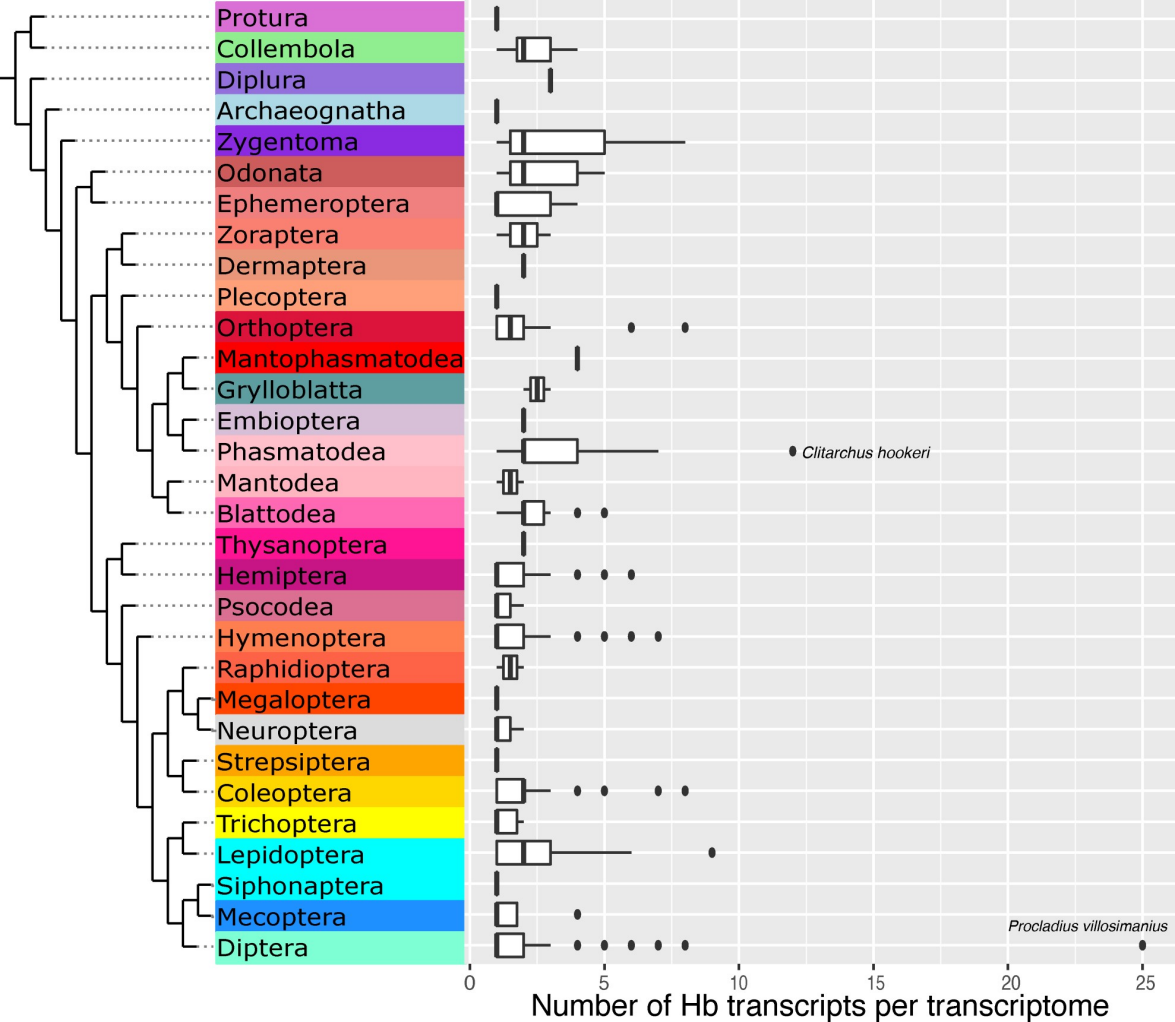
Hexapod phylogeny with transcripts per transcriptome per order. Box and whisker plot of the number of hemoglobins in each transcriptome, sorted by order. Box represents interquartile range with bar at the median value, whiskers indicate 1.5 x interquartile range, dots are outliers. Note, for example, *Procladius villosimanus*, a chironomid midge known to have multiple hemoglobin genes. Phylogeny after Misof et al. [50].

Expressed hemoglobins were located in 681 of 845 transcriptomes, representing 606 species. Although the levels of transcription of a gene do not necessarily dictate how much protein is synthesized[46–48], the broad presence of hemoglobin transcripts throughout hexapods strongly indicates protein expression. While specimen sampling was concentrated in certain orders (Diptera, Hymenoptera, Coleoptera, and Lepidoptera), the analysis uncovered hemoglobins across Hexapoda, with at least one species in every hexapod order possessing transcripts (Table 2). Many species expressed more than one hemoglobin sequence (Fig 2, S4 File)–which is unsurprising since insects possessing multiple hemoglobin genes are already known [15,21,49]. A total of 1333 unique hemoglobin transcripts were found.

**Table 2:** NCBI hexapod transcriptomes.

Order	Transcriptomes	Transcriptomes with Hbs	Transcriptomes with no Hbs	Species	Species with Hbs	Species with NO Hbs
Protura	1	1	0	1	1	0
Collembola	8	8	0	7	7	0
Diplura	2	1	1	2	1	1
Archaeognatha	2	2	0	2	2	0
Zygentoma	3	3	0	3	3	0
Odonata	7	7	0	7	7	0
Ephemeroptera	5	5	0	5	5	0
Zoraptera	2	2	0	2	2	0
Dermaptera	3	3	0	2	2	0
Plecoptera	4	3	1	4	3	1
Orthoptera	20	16	4	16	13	3
Mantophasmatodea	1	1	0	1	1	0
Grylloblattodea	2	2	0	2	2	0
Embioptera	2	2	0	2	2	0
Phasmatodea	21	21	0	18	18	0
Mantodea	3	2	1	3	2	1
Blattodea	12	10	2	9	8	1
Thysanoptera	5	3	2	5	3	2
Hemiptera	78	57	21	55	43	12
Psocoptera	2	2	0	2	2	0
Phthiraptera	1	1	0	1	1	0
Hymenoptera	284	257	27	266	248	18
Raphidioptera	3	2	1	3	2	1
Megaloptera	3	2	1	3	2	1
Neuroptera	7	7	0	7	7	0
Strepsiptera	2	1	1	2	1	1
Coleoptera	71	54	17	56	44	12
Trichoptera	6	6	0	6	6	0
Lepidoptera	131	87	44	90	64	26
Siphonaptera	4	3	1	4	3	1
Mecoptera	4	4	0	4	4	0
Diptera	146	106	40	126	97	29
Totals:	845	681	164	716	606	110

Hexapod transcriptomes per order available from NCBI TSA database, showing number with and without hemoglobins. Note that all orders have at least one hemoglobin-containing transcript. See text regarding explanations for putative lack of hemoglobins in some species. Note that the number of transcriptomes is greater than the number of species for several orders as some species were represented by multiple transcriptomes in the analysis.

### Source data—sampling of orders, rejected transcriptomes, contaminated taxa

From the set of 845 transcriptomes, 164 were found to not contain hemoglobins and hemoglobin-like sequences, so we sought to examine this apparent lack. For 36 of these Hb-less transcriptomes, a second transcriptome of the same species contained a hemoglobin transcript, indicating the species indeed contained an expressed hemoglobin. Many of these "double entries" were an updated, larger transcriptome from a smaller, older entry. Sequences located by the search as possible hemoglobins but containing fewer than 80 residues were considered to be unreliable, and this accounted for 16 transcriptomes. The remaining 112 transcriptomes were spread across 12 orders. Each of these 12 orders was represented by another transcriptome that contained hemoglobins; the absence of hemoglobin transcripts in these 112 transcriptomes did not influence the coverage of orders.

The size and quality of the assembled transcriptome was likely a factor in the success of locating hemoglobin. From the remaining 112 transcriptomes, 14 were significantly smaller, by more than an order of magnitude, than the average dataset in both contig and base pair count, and most likely incomplete. An additional 9 transcriptomes were assembled using Roche 454 sequencing, whereas the majority of transcriptomes in the study were assembled using Illumina. It is possible that novel sequences may be missed in 454-sequenced transcriptomes, since this technique has lower throughput.

To address the lack of hemoglobins in the remaining 89 transcriptomes, sample data submitted with each transcriptome's entry in NCBI was reviewed. Hemoglobin is not universally expressed across all tissue types and developmental stages, so targeted sampling used in many studies, such as specific tissues or developmental stages, could affect the presence or absence of hemoglobin transcripts (e.g., [16,27,51]). For example, hemoglobin expression in chironomids is known to be primarily in larval stages, and certain hemoglobins in *Chironomus thummi* are expressed during particular larval instars. Annotations from the NCBI TSA database for each of the remaining transcriptomes were used when supplied, and when no information was submitted, original references were consulted when available.

Forty-seven transcriptomes focused on specific tissue types, such as testes, venom glands, salivary glands, venom itself, and even regurgitant. Although functions of hemoglobins vary widely, in insects it is likely that sampling specific tissues could affect the search for hemoglobin transcripts. Additionally, 13 transcriptomes were taken from specific developmental stages. Since our study is a broad comparison for hemoglobins, transcriptomes assembled from a wide array of developmental stages—egg, larva, pupa, and adult—were considered to be more reliable datasets.

We were unable to assess the apparent lack of hemoglobin in 29 transcriptomes by examining metadata from NCBI and reviewing supplied references. These transcriptomes were spread across the following nine orders: Diplura, Orthoptera, Hemiptera, Hymenoptera, Raphidioptera, Coleoptera, Strepsiptera, Lepidoptera, and Diptera. (Table 3). We could find no apparent ecological, phylogenetic, or other commonalities to taxa without expressed hemoglobin transcripts. Further studies should test these apparent absences, ideally using both experimental and bioinformatic approaches.

**Table 3:** Taxa with no Hb transcripts located.

Taxon	Prefix	Order	Family
Nicrophorus orbicollis	GGAA01	Coleoptera	Silphidae
Nicrophorus vespilloides	GDKQ01	Coleoptera	Silphidae
Campodea augens	GAYN02	Diplura	Campodeidae
Bombylius major	GATI02	Diptera	Bombyliidae
Verticia nigra	GGHV01	Diptera	Calliphoridae
Chaoborus flavidulus	GGBK01	Diptera	Chaoboridae
Toxorhynchites sp. Toxo	GGBL01	Diptera	Culicidae
Tripteroides aranoides	GGBM01	Diptera	Culicidae
Liogma simplicicornis	GEMK01	Diptera	Cylindrotomidae
Bixinia sp. RSM007	GGGY01	Diptera	Rhinophoridae
Tipula maxima	GACZ01	Diptera	Tipulidae
Sitodiplosis mosellana	GAKJ01	Diptera	Cecidomyiidae
Acanthosoma haemorrhoidale	GAUV02	Hemiptera	Acanthosomatidae
Diaphorina citri	GACJ01	Hemiptera	Liviidae
Murgantia histrionica	GECQ01	Hemiptera	Pentatomidae
Courtella sp. AD-2014	GBTH01	Hymenoptera	Agaonidae
Elisabethiella stueckenbergi	GBTW01	Hymenoptera	Agaonidae
Aphidius ervi	GFLW01	Hymenoptera	Braconidae
Diaeretus essigellae	GBWM01	Hymenoptera	Braconidae
Orussus abietinus	GAUJ02	Hymenoptera	Orussidae
Orussus unicolor	GBTS01	Hymenoptera	Orussidae
Megaphragma amalphitanum	GFME01	Hymenoptera	Trichogrammatidae
Acanthopteroctetes unifascia	GENP01	Lepidoptera	Acanthopteroctetidae
Agathiphaga queenslandensis	GENX01	Lepidoptera	Agathiphagidae
Pseudopostega quadristrigella	GEOT01	Lepidoptera	Opostegidae
Plutella xylostella	GFRV01	Lepidoptera	Plutellidae
Locusta migratoria	GEZB01	Orthoptera	Acrididae
Raphidia ariadne	GACX01	Raphidioptera	Raphidiidae
Mengenilla moldrzyki	GACY01	Strepsiptera	Mengenillidae

The NCBI data was generally considered to be accurate and reliable, but an interesting example of contamination was uncovered. During the phylogenetic analysis (discussed below), a single sequence from the lacewing *Pseudomallada prasinus* (Neuroptera: Chrysopidae) was found to fall inside a clade of parasitoid wasps from the Braconidae and Ichneumonidae (See S1 Fig for the full amino acid cladogram). It is highly unlikely that the lacewing possesses hemoglobins similar in sequence to wasps; in fact, this transcriptome contains a second hemoglobin sequence that clusters with other Chrysopidae sequences. The most likely explanation is that the selected *P*. *prasinus* specimen harbored an endoparasitoid wasp larva (wasps of the families Eupelmidae and Perilampidae, for example, are known to be parasitoids of Chrysopidae). This brings up a few interesting points, including the possible transcriptome contamination of other parasitized specimens, and the apparent presence of hemoglobin in the wasp egg or larva.

### *Insectahemoglobins (IHbs)*—establishing homology and defining orthologs

As discussed, hemoglobin is present in a wide spectrum of life. The predominant functions of hemoglobins are enzymatic. Oxygen transport is a specialized development that accompanied the evolution of metazoans [19]. Hemoglobin sequences have diverged significantly to accommodate various roles [1], with many other functions likely to be uncovered (e.g., [4], [27]). Indeed, compared to vertebrate hemoglobins, those of invertebrates show much greater sequence and structural variation [1], perhaps just a reflection of the enormous diversity of the latter in terms of species numbers, body plans, physiology, and life histories.

**Fig 3:**
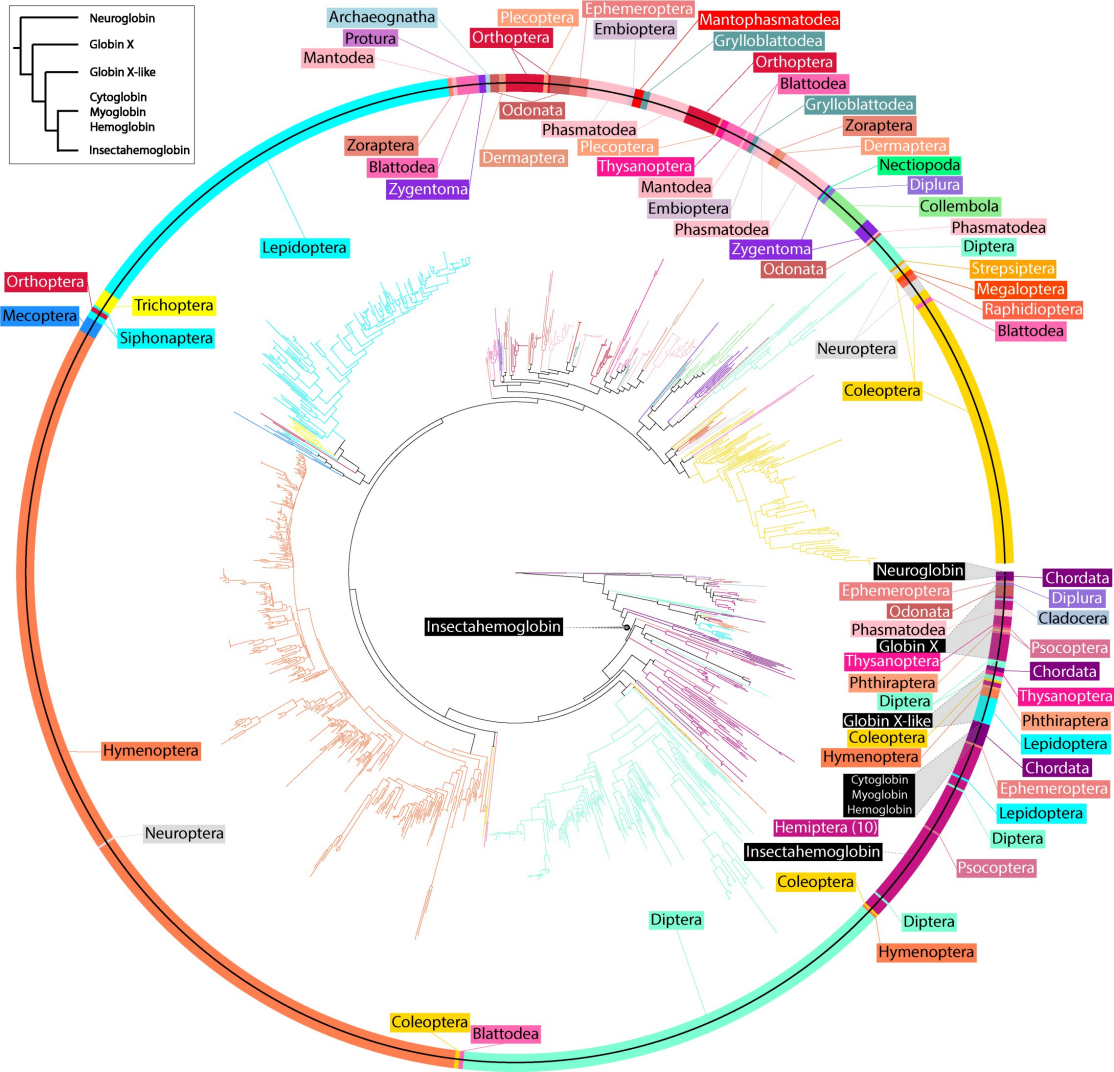
Circular hemoglobin gene tree of all 1382 coding sequences produced with maximum likelihood inference, denoting relationships of different globin types across chordates and the 32 hexapod orders. Colors are order specific, the same as used in Fig 2. Relationships among orders roughly correspond with the current understanding of hexapod relationships, but not directly due to the presence of transcripts from globins X and X-like and those resulting from gene duplications of hemoglobins (i.e., corresponding to *D*. *melanogaster* globins 1, 2, and 3). A simplified depiction of the globin relationships in the circular tree is presented in the top left inset.

The three hemoglobins of *Drosophila melanogaster* have been characterized in great detail fairly recently (e.g., [14,20,21,27]). The function of its globin-1 gene is likely to be for respiration and its glob-2 and 3 genes for male reproduction and spermatogenesis, so these have served as templates for finding orthologous genes in other taxa. According to primary sequence, including the presence of conserved sites (Figs 1 and S2 and S4 and S5 Files) and secondary structure, the majority of hexapod hemoglobins appear to be homologs of the *D*. *melanogaster* globin-1 gene (and other insect glob1 genes that have been described, sensu Table 1) or similar to the hemoglobin-like globins of Blank and Burmester [52] and Burmester et al. [27]. Although the globin-1 gene in brachyceran Diptera (including *D*. *melanogaster*) has an abbreviated N-terminal region before the first alpha-helix, this appears to be a derived condition, while most other hexapod globin-1 copies possess a longer N-terminal region. From our analyses of transcriptomes, it is unclear if the globin-2 and globin-3 copies in *D*. *melanogaster* are present in other insects as well. Many of the clades indicative of gene copies could represent independent acquisitions of a globin-2/3 function in various orders, and as has been shown in avian hemoglobins [42], similar biochemical functions can be acquired in independent ways. Particularly if androglobins [53] have been lost in many insect groups, as has been hypothesized for *D*. *melanogaster* [27], then globin-2/3 functions may have been independently acquired in several insect groups in compensation. As Gleixner et al. [51] demonstrated, the *D*. *melanogaster* globin-2/3 genes are quite divergent and have evolved at faster rates following duplication. Congruent with their findings, a small clade of dipterans (including *D*. *melanogaster*) apparently possessing globins-2/3 appear distant from the larger globin-1 clade in the cladogram (Figs 3 and S1) and may be a result of long-branch attraction. Interestingly, this hemoglobin 2/3 clade, in addition to *D*. *melanogaster*, contains only some mosquitoes, taxa that are not at all closely related within the Diptera. Within the monophyletic clade of hexapod hemoglobins, termed here ***insectahemoglobins (IHBs)***, the appearance of orders belonging to various hexapod clades (e.g., Entognatha, Palaeoptera, Polyneoptera) into two distinct parts of the tree indicate that transcripts are derived from at least two or more insectahemoglobin genes (as is the case for several orders that appear several times in separate parts of the tree, e.g., Zygentoma, Odonata, Blattodea, Phasmatodea, Hemiptera, some Coleoptera and Diptera) (S2 Fig). Analogous to the appearance of glob2-3 in Diptera, gene duplication events appear rather sporadic and could be specific to the order, family, or perhaps lower taxonomic levels. Distinct globin clades correspond to Blank and Burmester’s [52] globin X and globin X-like groupings (Figs 3 and S1). As the cladogram was estimated using transcripts from several gene copies, discrepancies with established hexapod phylogeny were expected (S3 Fig). Judging from localization predictions, certain phylogenetic discrepancies (e.g., Hemiptera adjacent to Diptera) likely also result from homoplasy due to convergent functions. However, basic relationships were conserved surprisingly well among groups, particularly within orders that likely contain only insectahemoglobin transcripts and their splice variants (S4–S6 Figs).

Although transcriptomes were not available for all of the species with previously described hemoglobins (Table 1), we recovered nearly all hemoglobin sequences for those which were, including a sequence likely from a novel gene copy of the *Bombyx mori* globin-1. The few cases of missing sequences from described hemoglobins appears to be attributable to the reasons mentioned above for missing hemoglobin sequences in various transcriptomes.

**Fig 4:**
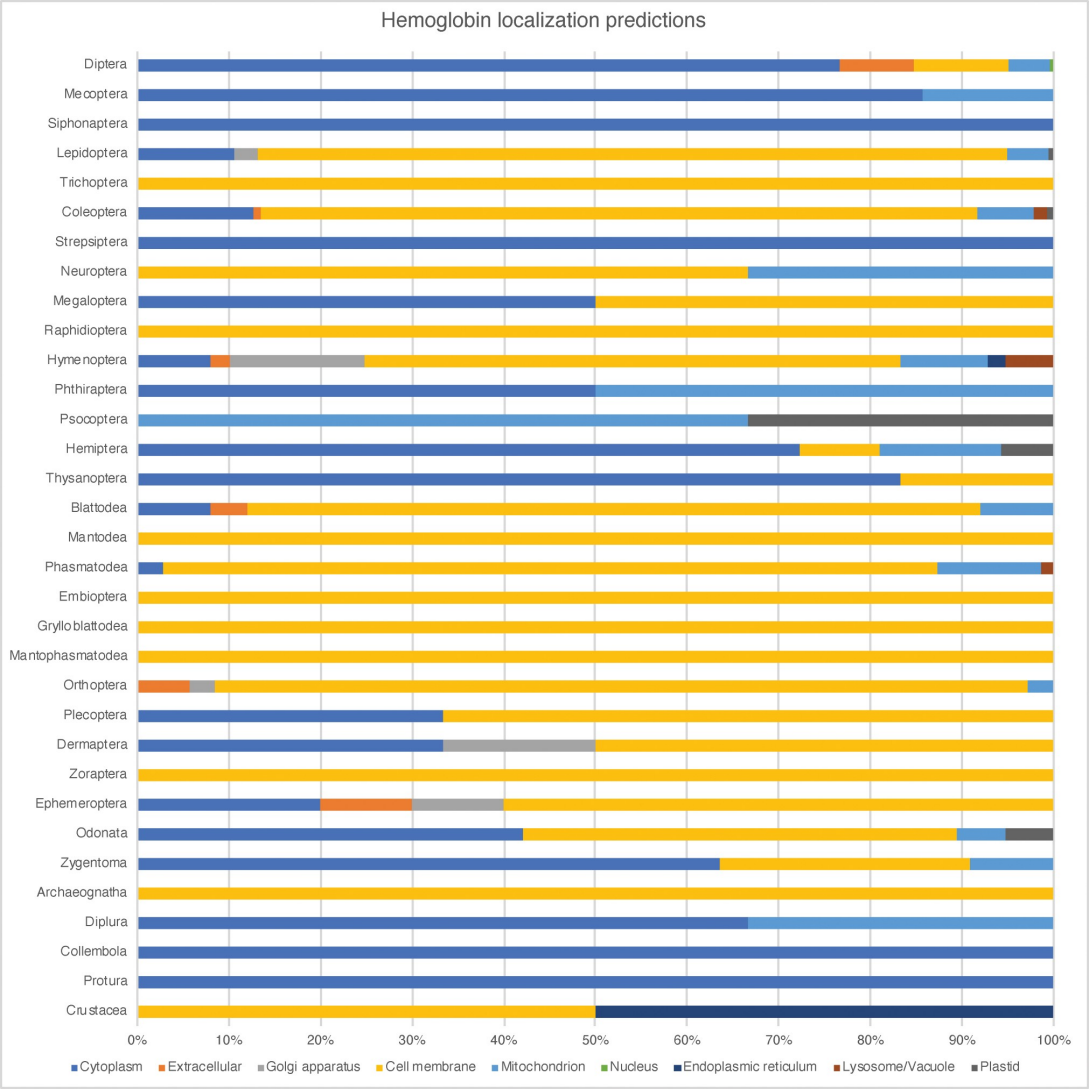
Localization predictions. Stacked bar plot of DeepLoc results, displaying globin localization predictions by order (S6 File for full results on all taxa). The majority of hexapod globins are intracellular and localized to the cell membrane (yellow), with many also found in the cytoplasm (dark blue) followed by the mitochondria (light blue).

We further classified some hexapod globins by incorporating into the analyses globins annotated in NCBI as hemoglobins, cytoglobins, neuroglobins, and even one supposed myoglobin (Figs 4 and S1 Fig). While the sequence terminology in such cases probably is derived from auto-annotated pipelines and inappropriate, the majority of the cytoglobins and neuroglobins actually fall within the globin X and X-like clades. Additionally, secondary structure was estimated for all globin sequences for visual comparison (Fig 1 and S3 Fig, S3 and S4 Files). Many groupings within and among insect orders corresponded to similarities in secondary structure. Structural differences within these groupings is probably due to the presence of additional gene copies or splice variants in various taxa. For instance, as mentioned the brachyceran Diptera possess a fairly abbreviated IHb with a short N-terminal domain (Fig 1 and S3 Fig). Lepidoptera possess a rather uniform IHb with a long N-terminal region, sometimes containing a short alpha-helix, possibly indicating different splice variants or stemming from different gene copies. Polyneopterans (earwigs, grasshoppers, roaches [including termites], mantises, and others) show some variation in their IHb but mostly have a long N-terminal region with a short alpha-helix. Coleoptera mostly show a long N-terminal domain containing loops, and Hymenoptera have IHb copies also with a long N-terminus containing either only loops or a short alpha-helix. The IHbs in Hemiptera are perhaps most variable in secondary structure in possessing transcripts with N-terminal domains of various lengths and alpha-helices of various lengths.

These patterns in IHb secondary structure among hexapod orders roughly correspond to those seen in our cell-localization predictions, in which the majority of dipteran and hemipteran IHbs are localized in the cytoplasm, while the majority of those in Hymenoptera, Lepidoptera, and Coleoptera are localized to cell membranes (Fig 4 and S6 File). Fewer IHbs are restricted to the Golgi apparatus, mitochondria, or which are extracellular.

To characterize protein structural differences, protein structure predictions were generated for all hemoglobins using I-TASSER and protein alignments estimated for each hemoglobin against all others using TM-align (S7 Fig and S7 and S8 Files). Generally, in congruence with the phylogenetic results of this study (including current data on hexapod phylogeny) and as noted above, IHb proteins were most similar (according to TM-score) within Hymenoptera and Diptera, closely followed by Coleoptera and Lepidoptera. Amphiesmenopteran (Lepidoptera and Trichoptera) scores were highest in comparison to the Antliophora (Diptera, Siphonaptera, and Mecoptera), the two groups comprising the Panorpida, and polyneopteran TM-scores were highest among all groups outside of Holometabola excepting Hymenoptera.

### Evolution of hexapod hemoglobins

Recent studies have hypothesized that hemoglobins may be present in most or all hexapods. Here we demonstrate this is the case and it involves a complex evolutionary history of gene duplication and several globin types (globin X, globin X-like, and insectahemoglobin). The relationships presented here among these globin types are congruent with previous studies (e.g., [52], [54–56]). As discussed by Blank and Burmester [52], globin X (and likely globin X-like) may not be involved in respiration. Our results show a scattered sampling of insect taxa in these two globin clades (Fig 3 and S1 Fig), containing transcripts that are membrane bound, cytoplasmic, and localized to mitochondria, probably indicative of various functions. Adjacent to the chordate myoglobins, cytoglobins, and hemoglobins, the remaining hexapod clades fall within the monophyletic insectahemoglobins, the majority derived from glob1-like gene copies. The phylogenetic location of IHbs suggests a structural convergence to the three chordate globins above. These results are consistent with previous studies that postulate independent acquisitions of hemoglobin function from early neuroglobin precursors through gene duplication and co-option [52]. This phenomenon may explain the widespread occurrence of hemoglobins in various kingdoms and phyla. The presence of key amino acids in IHbs indicates an ability to bind O_2_, but whether their sole function is respiratory is uncertain. Because of the divergence of the *D*. *melanogaster* glob2/3 genes and the current uncertainty in taxon distribution of androglobins in hexapods, it is unclear which other hexapods (if any) have independently evolved gene copies with glob2/3-like functions among the IHbs identified in this study. It therefore is possible that some fraction of the IHb genes are copies with glob2/3-like functions. Indeed, the current demonstration of hemoglobin expression in all hexapods leads to questions regarding relations to life histories (e.g., immatures versus adults, aquatic versus terrestrial, high versus low altitude), development, or various metabolically expensive processes such as flight, running, and swimming. It is difficult to determine any possible relationships of hemoglobin presence/transcript number to tracheal system architecture, which was the impetus for this study. It is possible that hemoglobins are common to nearly all life to support a respiratory role. Perhaps in insects, IHbs have a role in sequestration and intracellular transport and metabolism of respiratory gases, regardless of the tracheal architecture. The novel hemoglobin found in Remipedia in this study suggests IHbs may also be more prevalent in Arthropoda than previously recognized. Given the ubiquity of IHbs throughout hexapods, and their multiple forms (some proven to be important in respiration), the assumption that hexapod respiration is an entirely mechanical process involving just gaseous diffusion through tracheae probably needs to be abandoned. Future studies will need to address Hb functions in various taxa in attempting to define paralogs within the IHbs and to make broad-scale comparisons in the context of hexapod evolution. These analyses and results demonstrate the utility of large-scale genomic and transcriptomic computational analyses in guiding future experimental studies that will, in this case, probe the tissues and intracellular locations of hemoglobins and their functions.

## Materials and methods

Custom tools were developed in Python [57] and R [58] for data gathering, searches, analyses, and visualizations. Python versions 3.6.1 and 3.7.2 were used for scripting, in conjunction with BioPython versions 1.69 and 1.72 [59]. R version 3.5.1 was used within R Studio 1.1.453 [60]. The Supplementary Information contains implementation details for all scripts used in the study and a more comprehensive discussion of the workflow.

### Searching for hemoglobins

Candidate transcriptomes were obtained from the NCBI Transcriptome Shotgun Assembly (TSA) database [44]. Using online search tools, 845 taxa were identified, containing specimens from 29 orders of Insecta and 3 orders of Entognatha (S1 File). All transcriptomes were downloaded to local storage for analyses performed on-site at the American Museum of Natural History. BLAST searches and subsequent data analyses were run locally on a 16-node high-performance computing cluster using 256 cores. Although jobs were conducted in parallel as much as possible, more than 4 months of wallclock time was used in the analysis.

Using tblastx [61], candidate hemoglobin sequences were identified by comparing eight established arthropod (mostly insect) hemoglobins (S1 Table) against the transcriptomes of all 845 taxa. Although more computationally expensive than other BLAST searches, as all reading frames are checked, tblastx is effective at locating more conserved regions, and can be useful in locating novel genes in insect transcriptomes. Sequences with an Expect value greater than 0.01 were rejected.

All matched sequences were further verified and filtered using blastp to compare against the complete non-redundant (nr) NCBI protein database [62]. Sequences with high Expect values (greater than 0.01) and not matching a keyword search of 'globin' were discarded. Using the verified matches, which were occasionally "fragments", full coding sequences were extracted from the local transcriptome databases using custom-developed tools. Both nucleotide and amino acid datasets were constructed.

### Homology assessment

To properly identify homologies with established hemoglobins, 48 known globin sequences (see S2 Table) sampled from across Hexapoda and Chordata were included in a multiple sequence alignment. In addition to hemoglobins, previously annotated neuroglobins and cytoglobins were obtained from NCBI and included to better annotate the phylogenetic analysis and determine similarities to non-hemoglobin globins. To obtain a proper alignment, 92 short sequences consisting of fewer than 80 residues were removed. In addition, 29 sequences were hand-edited to truncate extraneous residues upstream of the start codon that were included due to incorrect automatic ORF determinations, and 10 additional sequences were removed as they were unable to be properly aligned. Removal of these sequences did not result in the reduction of representation across hexapod orders. The final dataset contained 1333 candidate hemoglobin sequences and the 48 globins from S2 Table, for a total of 1381 sequences. Alignments and visualizations of the 60-sequence subset (S5 File) chosen as "exemplars" used AliView [63], MAFFT [64], and the ETE toolkit [65].

The amino acid dataset was aligned using MAFFT 7.310 with the Needleman-Wunsch algorithm and 1000 cycles of iterative refinement. RevTrans 1.4, a codon-based aligner, was used for the nucleotide dataset [66]. The resulting alignments were 865 positions for amino acids (S4 File) and 2463 for nucleotides.

### Phylogenetic analyses

Hemoglobin gene trees were constructed using RAxML 8.2.11 [67]. Amino acid coding sequence relationships were determined using the LG substitution model with empirical base frequencies, optimization of substitution rates with a GAMMA model of rate heterogeneity, and 1000 bootstrap replicates with a rapid bootstrap analysis. For the nucleotide analysis, GTR with optimization of substitution rates and a GAMMA model of rate heterogeneity was used (alpha parameter was estimated), along with an estimate of proportion of invariable sites.

### Structure prediction and alignment

Secondary structure prediction on all 1381 sequences used a local installation of PSIPRED 4.0 [68] and the Uniref90 [69] database. Additionally, full three-dimensional structure models were computed using both a local installation of I-TASSER 5.1 and the on-line I-TASSER server [70,71]. Tertiary structure alignments were performed for every predicted model against every other model using TM-align [72].

### Localization predictions

Cellular localization prediction was achieved using the online DeepLoc 1.0 server.[73] Results were processed using custom Python scripts and visualizations created with Excel. Predictions were further verified using the NCBI Conserved Domains Database online search.[74]

### Other tools

Tree visualizations were composed using the ETE toolkit.[65] Conservation of functionally important residues was calculated using Jalview 2.10.5 [45]. AliView 1.24 was used for viewing and editing alignments and sequences. Mesquite version 3.51 was used for viewing alignments, formatting files for use by RAxML and other programs, and previewing trees [75]. Tanglegrams were rendered using the ETE toolkit and Abobe Illustrator CC 2018 (Version 22.1). Heat maps from tertiary alignments were produced using Python via JupyterLab and the libraries Pandas, Seaborn, MatplotLib, and Numpy from the SciPy toolkit [76,77].

## Supporting information

S1 Fig Amino acid globin tree with chordate globins. Cladogram of all globin transcripts, including 18 chordate globins, from amino acid analysis in RAxML. Bootstrap values labeled at nodes. Colored tags near various taxa in tree indicate locations of previously known hemoglobin sequences included to assist in homology assessment (S2 Table)[‘globin’, red]. Hemoglobin types labeled as belonging to globin X and X-like (according to Blank and Burmester [52]) or insectahemoglobin lineages (i.e., corresponding to D. melanogaster globins 1, 2, and 3).
(PDF)

S2 Fig Insectahemoglobin gene tree, rooted on Remipedia. (PDF)

S3 Fig Globin gene tree and phylogeny of insects. Tanglegram comparing the full globin tree (left), resulting from the nucleotide analysis of all hexapod hemoglobin transcripts in this study (summarized by order), and the phylogeny of Hexapoda (right) (redrawn from Misof et al. [50]). Note that phylogeny includes insectahemoglobins and X and X-like globins. Relative topological incongruence between gene (globin) and taxon trees is indicated by number of overlapping lines connecting clades.
(PDF)

S4 Fig Diptera hemoglobins and corresponding Diptera family phylogeny. Tanglegram comparing the tree of Diptera hemoglobins (left) and the phylogeny of Diptera (right) (redrawn from Wiegmann et al. [78]). IHb relationships are derived from the full taxon amino acid analysis.
(PDF)

S5 Fig Hymenoptera hemoglobins and corresponding Hymenoptera family phylogeny. Tanglegram comparing the Hymenoptera insectahemoglobin tree (left) and the phylogeny of Hymenoptera (right) (redrawn from Peters et al. [79]). IHb relationships are derived from the full taxon amino acid analysis.
(PDF)

S6 Fig Lepidoptera hemoglobins and corresponding lepidoptera family phylogeny. Tanglegram comparing the Lepidoptera insectahemoglobin tree and the phylogeny of Lepidoptera (redrawn from Mitter et al. [80]). IHb relationships are derived from the full taxon amino acid analysis.
(PDF)

S7 Fig Globin structural similarity. Heatmap produced from protein prediction structure alignments. Structure predictions from I-TASSER were used to compare every globin against every other globin using TM-align. Comparisons are not transitive–above the diagonal, the row element taxon is the template protein, below the diagonal, the column element taxon is the template protein. Values along the diagonal are 1.0. Nearly all TM-align values were greater than 0.5, the threshold for structural similarity [72]. Taxa are listed in phylogenetic order after Misof et al. [50]. Shade of green indicates similarity. White areas indicate missing structure predictions.
(PNG)

S1 Table Hemoglobin "target" genes used for searching. (DOCX)

S2 Table Established 'globin' genes used for alignments and phylogenetic analysis. (DOCX)

S1 File TSA Hexapoda transcriptome data table. (CSV)

S2 File Supplementary methods and materials. Workflow and scripting implementation details.
(DOCX)

S3 File Globin transcript multiple sequence alignment (amino acids). (FASTA)

S4 File Hemoglobin counts per taxon. (CSV)

S5 File Multiple sequence alignment of 60 exemplar hemoglobin amino acid sequences selected from across Hexapoda. (FASTA)

S6 File DeepLoc results for all globin sequences. (XLSX)

S7 File TM-align output of protein alignments between all hemoglobins. (XLSX)

S8 File TM-align output of protein alignments between hemoglobins of exemplar subset. (XLSX)
