# Supplementary figures and images for: Transcriptomes reveal expression of hemoglobins throughout insects and other Hexapoda
Source: PLoS One. 2020 Jun 5;15(6):e0234272. doi: 10.1371/journal.pone.0234272 (PMC7274415; doi:10.1371/journal.pone.0234272)

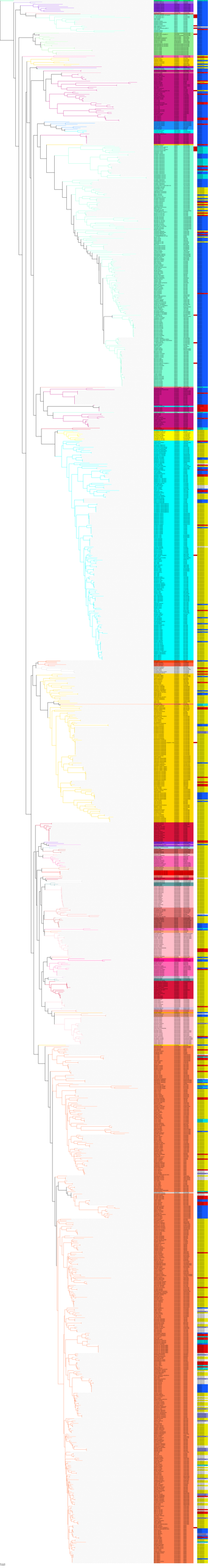

Supplement: S2 Fig — (PDF) [file pone.0234272.s002.pdf]

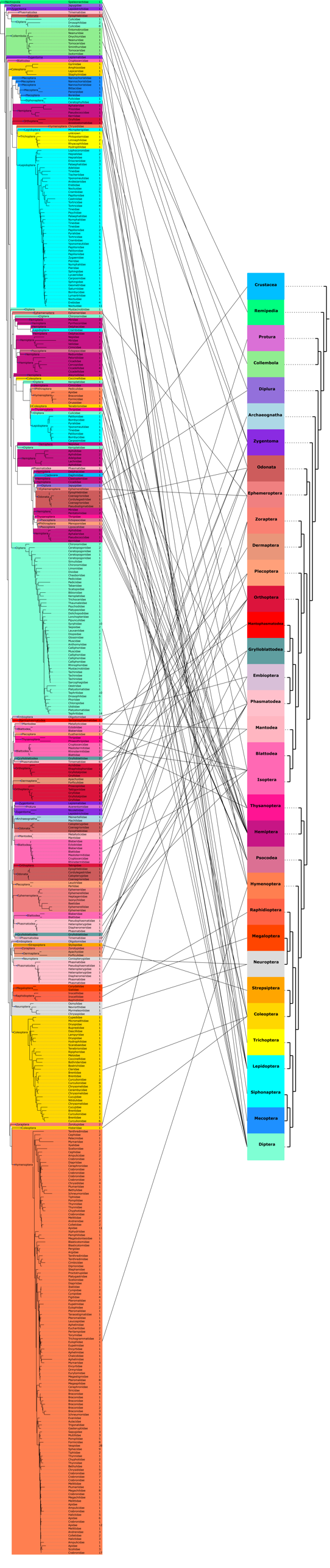

Supplement: S3 Fig — Tanglegram comparing the full globin tree (left), resulting from the nucleotide analysis of all hexapod hemoglobin transcripts in this study (summarized by order), and the phylogeny of Hexapoda (right) (redrawn from Misof et al. [50]). Note that phylogeny includes insectahemoglobins and X and X-like globins. Relative topological incongruence between gene (globin) and taxon trees is indicated by number of overlapping lines connecting clades. (PDF) [file pone.0234272.s003.pdf]

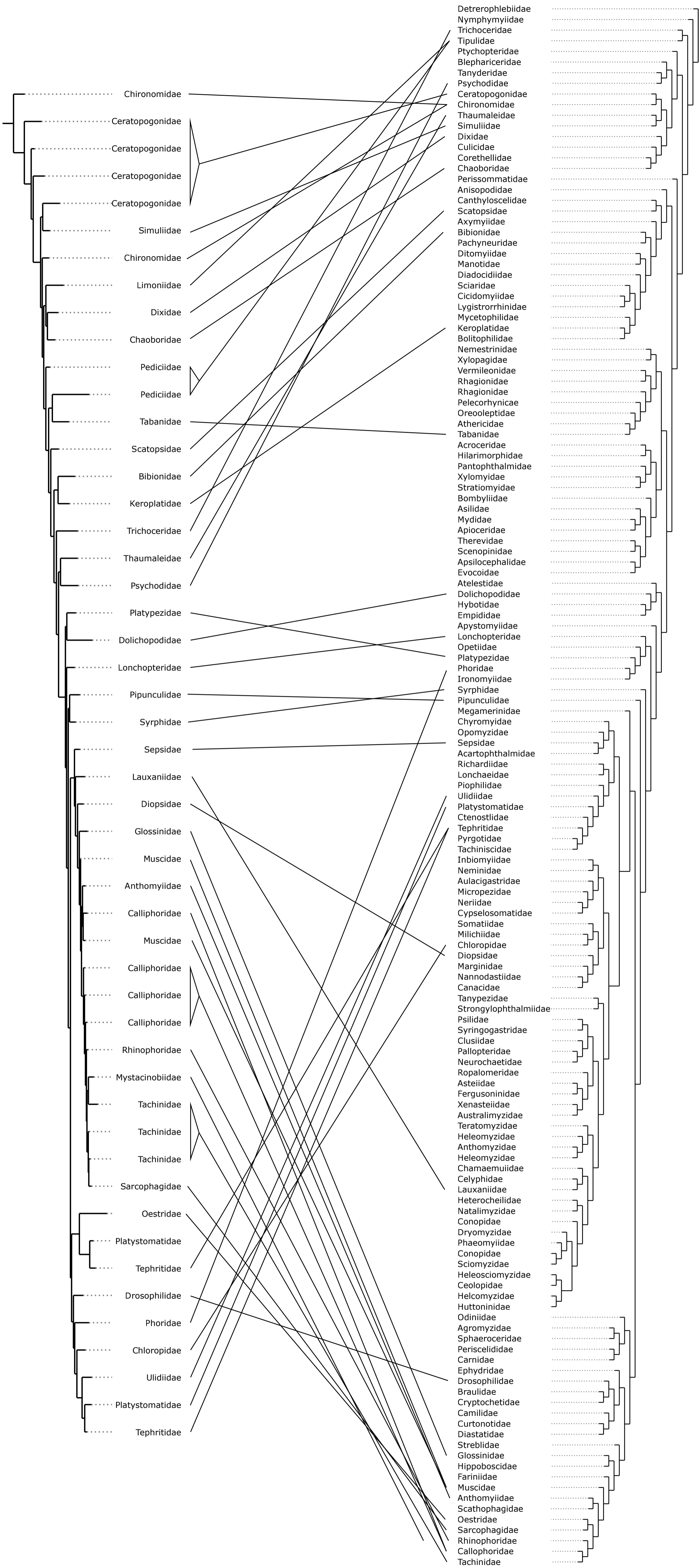

Supplement: S4 Fig — Tanglegram comparing the tree of Diptera hemoglobins (left) and the phylogeny of Diptera (right) (redrawn from Wiegmann et al. [78]). IHb relationships are derived from the full taxon amino acid analysis. (PDF) [file pone.0234272.s004.pdf]

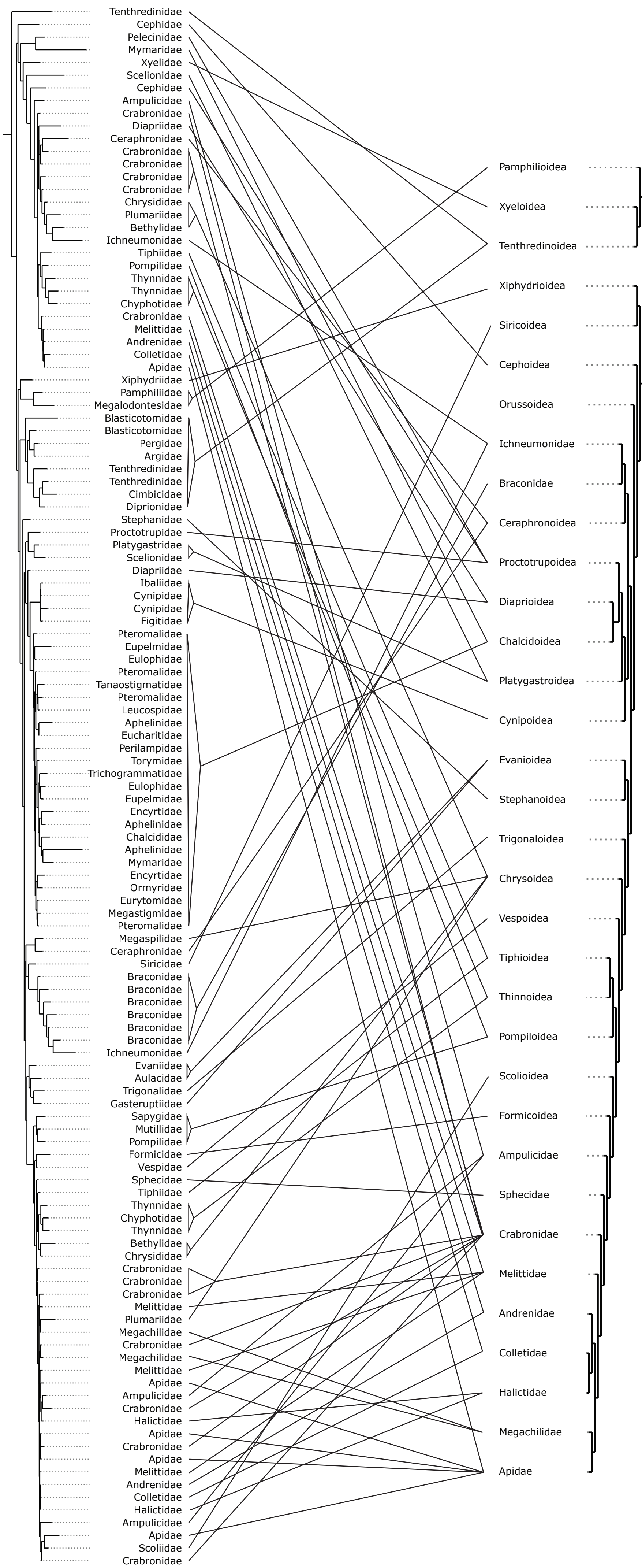

Supplement: S5 Fig — Tanglegram comparing the Hymenoptera insectahemoglobin tree (left) and the phylogeny of Hymenoptera (right) (redrawn from Peters et al. [79]). IHb relationships are derived from the full taxon amino acid analysis. (PDF) [file pone.0234272.s005.pdf]

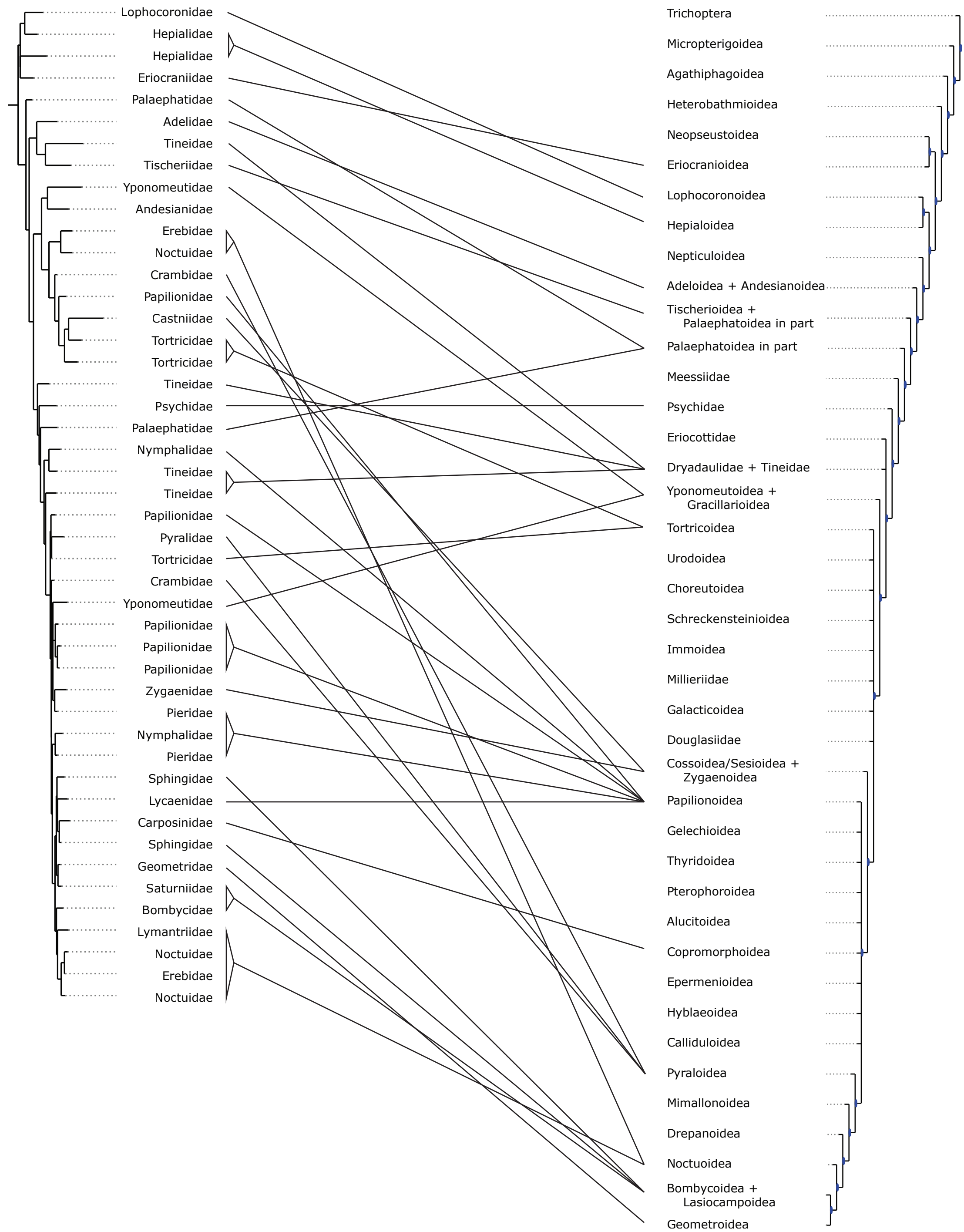

Supplement: S6 Fig — Tanglegram comparing the Lepidoptera insectahemoglobin tree and the phylogeny of Lepidoptera (redrawn from Mitter et al. [80]). IHb relationships are derived from the full taxon amino acid analysis. (PDF) [file pone.0234272.s006.pdf]
