# Supplementary material for: Transcriptomes reveal expression of hemoglobins throughout insects and other Hexapoda
Source: PLoS One. 2020 Jun 5;15(6):e0234272. doi: 10.1371/journal.pone.0234272 (PMC7274415; doi:10.1371/journal.pone.0234272)
Supplement: S1 Table — (DOCX) [file pone.0234272.s008.docx]

Table S1. Hemoglobin "target" genes used for searching.

| Taxon | Source and Identifier | Designation |
| --- | --- | --- |
| *Anisops notonectidae* | NCBI HE795534.1 | HbA gene |
| *Chironomus* sp. (gene cluster) | NCBI X56271.1 |  |
| *Culex quinquefasciatus* | NCBI XM_001847130.1 |  |
| *Daphnia* sp. | NCBI U67067.1 | Dhb1 |
| *Drosophila melanogaster* (gene cluster) | Flybase FBgn0027657 | Dmel_glob1 |
| *Drosophila melanogaster* | Flybase FBpp0082701 | Dmel_glob1 |
| *Drosophila melanogaster* (globin domain) | Flybase | Dmel_glob1 |
| *Gasterophilus intestinalis* | NCBI AF063938.1 | glob1 |
