# Supplementary material for: Transcriptomes reveal expression of hemoglobins throughout insects and other Hexapoda
Source: PLoS One. 2020 Jun 5;15(6):e0234272. doi: 10.1371/journal.pone.0234272 (PMC7274415; doi:10.1371/journal.pone.0234272)
Supplement: S2 Table — (DOCX) [file pone.0234272.s009.docx]

Table S2. Established 'globin' genes used for alignments and phylogenetic analysis. Note: the hemoglobin for *Speleonectes* (Remipedia) was determined in this study.

| Taxon | Gene | Designation | ID |
| --- | --- | --- | --- |
| *Drosophila melanogaster* | Hemoglobin | Dmel_glob1 | FBpp0082701 |
| *Drosophila melanogaster* | Hemoglobin | Dmel_glob2 | FBpp0290501 |
| *Drosophila melanogaster* | Hemoglobin | Dmel_glob3 | FBpp0078289 |
| *Gasterophilus intestinalis* | Hemoglobin | Chain A | PDB 2C0K |
| *Chironomus thummi* | Hemoglobin | Ctp HbVIIB-5 | CAA39714.1 |
| *Chironomus thummi* | Hemoglobin | Ctp HbV | CAA39718.1 |
| *Chironomus thummi* | Hemoglobin | Ctp HbY | CAA39718.1 |
| *Chironomus thummi* | Hemoglobin | globin W | AAA85483.1 |
| *Speleonectes cf. tulumensis* |  |  | JL117411.1 |
| *Daphnia magna* | Neuroglobin |  | JAM19901.1 |
| *Culex quinquefasciatus* | Neuroglobin |  | XP_001847170.1 |
| *Culex quinquefasciatus* | Hemoglobin |  | XP_001847182.1 |
| *Culex quinquefasciatus* | Globin |  | EDS33969.1 |
| *Aedes aegypti* | Neuroglobin |  | XP_021711312.1 |
| *Aedes aegypti* | Myoglobin |  | XP_001650222.1 |
| *Trachymyrmex cornetzi* | Neuroglobin |  | KYN11311.1 |
| *Tribolium castaneum* | Neuroglobin | (Predicted) | XP_015834431.1 |
| *Orussus abietinus* | Neuroglobin |  | XP_012284967.1 |
| *Pediculus humanus corporis* | Neuroglobin | Putative | XP_002426939.1 |
| *Bombyx mori* | Neuroglobin |  | XP_012546380.1 |
| *Bombyx mori* | Neuroglobin |  | XP_004926839.1 |
| *Papilio machaon* | Neuroglobin |  | KPJ17275.1 |
| *Papilio machaon* | Neuroglobin |  | KPJ17276.1 |
| *Papilio machaon* | Cytoglobin | Cytoglobin-1 | KPJ08246.1 |
| *Cimex lectularius* | neuroglobin-like |  | XP_014248500.1 |
| *Cimex lectularius* | neuroglobin-like |  | XP_014253735.1 |
| *Apis cerana cerana* | Cytoglobin | Cytoglobin-1 | PBC27039.1 |
| *Orchesella cincta* | Cytoglobin | Cytoglobin-2 | ODM89928.1 |
| *Bactrocera latifrons* | Cytoglobin | Cytoglobin-1 | JAI31664.1 |
| *Bombus terrestris* | Cytoglogin | Cytoglobin-2 | XP_003396832.1 |
| *Dendroctonus ponderosae* | Cytoglobin | Cytoglobin-1-like | XP_019758973.1 |
| *Homo sapiens* | hemoglobin chain A |  | pdb\|2W72\|A |
| *Danio rerio* | hemoglobin subunit alpha |  | NP_571332.3 |
| *Homo sapiens* | hemoglobin chain B |  | pdb\|2W72\|B |
| *Danio rerio* | hemoglobin subunit beta-1 |  | NP_571095.1 |
| *Homo sapiens* | cytoglobin |  | NP_599030.1 |
| *Danio rerio* | cytoglobin-1 |  | NP_694484.1 |
| *Danio rerio* | cytoglobin-2 |  | NP_001019395.1 |

(Table 2 continued on next page)

­

Table S2 (continued from previous page). Established 'globin' genes used for alignments and phylogenetic analysis.

| Taxon | Gene | Designation | ID |
| --- | --- | --- | --- |
| *Homo sapiens* | neuroglobin |  | NP_067080.1 |
| *Danio rerio* | neuroglobin |  | NP_571928.1 |
| *Homo sapiens* | myoglobin |  | NP_005359.1 |
| *Danio rerio* | myoglobin |  | NP_956880.1 |
| *Danio rerio* | x globin |  | NP_001012261.2 |
| *Acyrthosiphon pisum* | neuroglobin |  | XP_029346817.1 |
| *Branchiostoma floridae* | Gb1 hypothetical protein | BRAFLDRAFT_98913 | XP_002608549.1 |
| *Branchiostoma floridae* | Gb7 globin |  | CBL51553.1 |
| *Branchiostoma floridae* | Gb3 hypothetical protein | BRAFLDRAFT_99970 | XP_002610016.1 |
| *Branchiostoma floridae* | Gb12 hypothetical protein | BRAFLDRAFT_74222 | XP_002605405.1 |
