# Supplementary material for: Transcriptomes reveal expression of hemoglobins throughout insects and other Hexapoda
Source: PLoS One. 2020 Jun 5;15(6):e0234272. doi: 10.1371/journal.pone.0234272 (PMC7274415; doi:10.1371/journal.pone.0234272)
Supplement: S2 File — Workflow and scripting implementation details. (DOCX) [file pone.0234272.s011.docx]

# Supplementary Methods and Materials

Although the Materials and Methods were reported in the main body of the manuscript, this section is intended to be a detailed, standalone description of the workflow implemented to locate putative hemoglobin genes. As a result, some repetition between the two sections is required. Figures and Tables from the main body of the paper are referred to as 'Table 1', and so on, while they are referred to as 'Table S1' for tables present only in the Supplementary Information and associated data files.

## Custom scripting used in the analysis

A number of custom scripts were developed in Python. Scripts were used to semi-automatically download and organize data, reformat sequence files, submit jobs to the high-performance compute cluster, collate results, and visualize trees and alignments. Python versions 3.6.1 and 3.7.2 [1] were used for scripting, in conjunction with BioPython versions 1.69 and 1.72 [2]. R version 3.5.1 [3] was used within R Studio 1.1.453 [4]. Source code for scripts is available upon request.

## Obtaining data - Transcriptomes

Transcriptomes were identified from the NCBI Transcriptome Shotgun Assembly database by filtering for all available insect assemblies [5]. The results of this query, 845 listed transcriptomes, were downloaded to a csv file (lib/wgs_selector.csv) and placed under git revision control along with all Python and R scripts used in the analysis. Family and Order for every taxon in the list were added using a pre-generated taxonomy list [6] with a custom script (bin/find_lineages.py), creating the input list for all taxa used in the analysis (lib/transcriptomes_family_order.csv).

## Database preparation

Database searches and analyses were performed at the American Museum of Natural History's (AMNH) Sackler Institute for Comparative Genomics (SICG) bioinformatics computing cluster. All databases used were downloaded to local storage at SICG and BLAST runs were performed using a standalone BLAST installation using BLAST version 2.8.0+.

Using a custom script (bin/fetch_transcriptomes.py), transcriptomes were downloaded from the NCBI FTP site at ftp://ftp.ncbi.nlm.nih.gov and placed into a "Transcripts" directory containing separate sub-directories for each taxon, indexed by taxon name and prefix (e.g., Transcripts/Triarthria_setipennis_GAVA02). Using the list of 845 taxa, the script automatically downloaded, decompressed, and created BLAST databases using makeblastdb with the options '-dbtype nucl -hash_index -parse_seqids'.

## Searching for Hemoglobin Genes

Eight established arthropod hemoglobin gene sequences were obtained from NCBI [5] and Flybase [7] as nucleotide sequences, detailed in Table S1. These genes were used as query sequences to search for potential hemoglobin genes in every transcriptome. Locating potential insect hemoglobin genes was performed as follows:

1. Locate hemoglobin-like sequences in transcriptome. Compare several known invertebrate hemoglobin genes against each taxon's transcriptome (query_transcriptomes.py).
2. Verify these matches as putative hemoglobins by comparing against entire NCBI non-redundant (nr) database (bin/verify_transcriptome_results.py), filtering for keyword 'globin' in nr description and checking for Expect values <= 0.01 (bin/final_transcriptome_matches.py).
3. Extract coding sequences from transcriptome using putative hemoglobin matches. Sequences are extracted as both nucleotide and amino acid FASTA files.

Details of the implementation of each of these steps is discussed below.

## Database searching 1 - looking for hemoglobin-like sequences in insect transcriptomes

A custom script (bin/query_transcripomes.py) was created to run blast searches for every hemoglobin gene query against all 845 transcriptomes. tblastx was used with default command line options (e.g., -num_threads 32 -outfmt 10 ...) to search translated nucleotide databases with translated nucleotide queries. Although slightly more computationally intensive than other BLAST algorithms as all 6 possible reading frames are checked, tblastx was chosen to search for novel genes - previously unidentified possible hemoglobin sequences in insect transcriptomes, and as tblastx compares amino acid sequences, it is more effective at locating more conserved regions.

Results were stored in each taxon's transcriptome directory, sorted by query name, with one file per hemoglobin query (e.g. 'Transcripts/Osmia_cornuta_GAGH01/Osmia_cornuta_GAGH01-Anisops_Notonectidae_Hemoglobin.fasta.tblastx.out'). The following information was retrieved for each database match (detailed information on identifiers available from NCBI BLAST documentation):

| Abbrev | Designation |
| --- | --- |
| qseqid | Hb query sequence ID |
| sseqid | Subject sequence ID |
| nident | Number of identical matches |
| pident | Percentage of identical matches |
| length | Alignment length |
| qstart | Hb query start offset |
| qend | Hb query end offset |
| sstart | Subject start offset |
| send | Subject end offset |
| sseq | Subject sequence match (amino acid sequence) |
| qseq | Query sequence match (amino acid sequence) |
| evalue | Expect value for this match |

## Database searching 2 - verification of matches as possible hemoglobins

The sequences from this first step represent regions in the transcriptome that potentially match established hemoglobin genes. For the second step, validation of these sequences as hemoglobins, a two-phase "verification" was performed.

First, every sequence was compared against the complete non-redundant (nr) database [8] (downloaded as of June 2018 and used for standalone local searches). A custom script (bin/verify_transcriptome_results.py) was used to search nr with every matching sequence using blastp and default options. Results were stored in subdirectories sorted by query as NCBI XML formatted files, with one file for each match from searching hemoglobin genes against transcriptomes. Filenames were formatted to contain human-readable information regarding sequence identifiers and start and stop indices (e.g., Transcriptomes/Nomia_diversipes_GBWP01/Dm_glob1_CDS/FBpp0082701-gb|GBWP01020494.1|-1-126-248-373.xml). Results were limited to the first 500 hits for each sequence. Finally, the results from comparing the sequence against nr were verified by thresholding results to have an Expect value no greater than 0.01 and searching for the keyword 'globin' in the NCBI XML BLAST identifier description. Every sequence from the first step satisfying these criteria were stored as amino acid sequences in a FASTA file (Hb_transcriptome_matches.fasta) with the header for each sequence containing taxonomy information and identifiers useful in extracting transcript data from the taxon's transcriptome database. For example, matches from two transcripts of the transcriptome for *Procladius villosimanus* is shown:

>Procladius villosimanus-(Diptera:Chironomidae) Procladius_villosimanus_GGAY01:gb|GGAY01021822.1|,(366-13)

ILFAYFEKYPINQEKFPAFRNTPLLLLKGTPGFRSHASKIMNVFSCAIDGLDRENGLQEVAKIVAHVGEYHADRKITKRSMVEMREVLTEKLIEVCHLDDEAIQAWSNLLDIVWCIMF

>Procladius villosimanus-(Diptera:Chironomidae) Procladius_villosimanus_GGAY01:gb|GGAY01021822.1|,(369-16)

KILFAYFEKYPINQEKFPAFRNTPLLLLKGTPGFRSHASKIMNVFSCAIDGLDRENGLQEVAKIVAHVGEYHADRKITKRSMVEMREVLTEKLIEVCHLDDEAIQAWSNLLDIVWCIM

>Procladius villosimanus-(Diptera:Chironomidae) Procladius_villosimanus_GGAY01:gb|GGAY01021822.1|,(366-19)

ILFAYFEKYPINQEKFPAFRNTPLLLLKGTPGFRSHASKIMNVFSCAIDGLDRENGLQEVAKIVAHVGEYHADRKITKRSMVEMREVLTEKLIEVCHLDDEAIQAWSNLLDIVWCI

>Procladius villosimanus-(Diptera:Chironomidae) Procladius_villosimanus_GGAY01:gb|GGAY01021822.1|,(381-49)

NKGEKILFAYFEKYPINQEKFPAFRNTPLLLLKGTPGFRSHASKIMNVFSCAIDGLDRENGLQEVAKIVAHVGEYHADRKITKRSMVEMREVLTEKLIEVCHLDDEAIQAW

>Procladius villosimanus-(Diptera:Chironomidae) Procladius_villosimanus_GGAY01:gb|GGAY01021822.1|,(366-10)

ILFAYFEKYPINQEKFPAFRNTPLLLLKGTPGFRSHASKIMNVFSCAIDGLDRENGLQEVAKIVAHVGEYHADRKITKRSMVEMREVLTEKLIEVCHLDDEAIQAWSNLLDIVWCIMFD

>Procladius villosimanus-(Diptera:Chironomidae) Procladius_villosimanus_GGAY01:gb|GGAY01021822.1|,(366-34)

ILFAYFEKYPINQEKFPAFRNTPLLLLKGTPGFRSHASKIMNVFSCAIDGLDRENGLQEVAKIVAHVGEYHADRKITKRSMVEMREVLTEKLIEVCHLDDEAIQAWSNLLD

>Procladius villosimanus-(Diptera:Chironomidae) Procladius_villosimanus_GGAY01:gb|GGAY01021822.1|,(366-133)

ILFAYFEKYPINQEKFPAFRNTPLLLLKGTPGFRSHASKIMNVFSCAIDGLDRENGLQEVAKIVAHVGEYHADRKITK

>Procladius villosimanus-(Diptera:Chironomidae) Procladius_villosimanus_GGAY01:gb|GGAY01021821.1|,(362-9)

ILFAYFEKYPINQEKFPAFRNTPLLLLKGTPGFRSHASKIMNVFSCAIDGLDRENGLQEVAKIVAHVGEYHADRKITKRSMVEMREVLTEKLIEVCHLDDEAIQAWSNLLDIVWCIMF

>Procladius villosimanus-(Diptera:Chironomidae) Procladius_villosimanus_GGAY01:gb|GGAY01021821.1|,(365-12)

KILFAYFEKYPINQEKFPAFRNTPLLLLKGTPGFRSHASKIMNVFSCAIDGLDRENGLQEVAKIVAHVGEYHADRKITKRSMVEMREVLTEKLIEVCHLDDEAIQAWSNLLDIVWCIM

>Procladius villosimanus-(Diptera:Chironomidae) Procladius_villosimanus_GGAY01:gb|GGAY01021821.1|,(362-15)

ILFAYFEKYPINQEKFPAFRNTPLLLLKGTPGFRSHASKIMNVFSCAIDGLDRENGLQEVAKIVAHVGEYHADRKITKRSMVEMREVLTEKLIEVCHLDDEAIQAWSNLLDIVWCI

>Procladius villosimanus-(Diptera:Chironomidae) Procladius_villosimanus_GGAY01:gb|GGAY01021821.1|,(377-45)

NKGEKILFAYFEKYPINQEKFPAFRNTPLLLLKGTPGFRSHASKIMNVFSCAIDGLDRENGLQEVAKIVAHVGEYHADRKITKRSMVEMREVLTEKLIEVCHLDDEAIQAW

>Procladius villosimanus-(Diptera:Chironomidae) Procladius_villosimanus_GGAY01:gb|GGAY01021821.1|,(362-6)

ILFAYFEKYPINQEKFPAFRNTPLLLLKGTPGFRSHASKIMNVFSCAIDGLDRENGLQEVAKIVAHVGEYHADRKITKRSMVEMREVLTEKLIEVCHLDDEAIQAWSNLLDIVWCIMFD

>Procladius villosimanus-(Diptera:Chironomidae) Procladius_villosimanus_GGAY01:gb|GGAY01021821.1|,(362-30)

ILFAYFEKYPINQEKFPAFRNTPLLLLKGTPGFRSHASKIMNVFSCAIDGLDRENGLQEVAKIVAHVGEYHADRKITKRSMVEMREVLTEKLIEVCHLDDEAIQAWSNLLD

>Procladius villosimanus-(Diptera:Chironomidae) Procladius_villosimanus_GGAY01:gb|GGAY01021821.1|,(362-129)

ILFAYFEKYPINQEKFPAFRNTPLLLLKGTPGFRSHASKIMNVFSCAIDGLDRENGLQEVAKIVAHVGEYHADRKITK

Note that this is a small subset for this taxon, showing the results from only two transcripts. For the complete dataset of 845 transcriptomes, a total of 5,769 potentially hemoglobin-matching sequences were identified.

## Database searching 3 - extract coding sequences

As shown, due to the nature of the sequence searching, a given transcript may have multiple sequence matches. Properly identifying homologous regions requires locating the full coding sequence from each transcript. To locate and extract coding sequences from the set of database matches, a custom script (bin/get_transcript_cds_from_alignment.py) was used to retrieve the complete transcript from the local database and search for the correct CDS corresponding to the verified hemoglobin matches from the previous step. All six possible translations were string-compared against the original BLAST matches for each transcript to determine the correct reading frame. Python regular expressions isolated the CDS, and BioPython pairwise alignment was used to verify the final CDS against the original matched sequence. As mentioned, multiple sequence matches from the previous step that matched with the same transcript were "collapsed" such that each matching transcript was output once (Hb_transcriptome_CDS.fasta).

For example, continuing with *Procladius villosimanius*, the complete CDS is shown below with the matched sequence highlighted:

>Procladius_villosimanus__GGAY01021822.1

MKTLTPEQVAIIKRTWEPIQSDHFNKGEKILFAYFEKYPINQEKFPAFRNTPLLLLKGTP

GFRSHASKIMNVFSCAIDGLDRENGLQEVAKIVAHVGEYHADRKITKRSMVEMREVLTEK

LIEVCHLDDEAIQAWSNLLDIVWCIMFDCFD

>Procladius_villosimanus__GGAY01021821.1

MGNVITNIQKKIHPSENIPMKTLTPEQVAIIKRTWEPIQSDHFNKGEKILFAYFEKYPIN

QEKFPAFRNTPLLLLKGTPGFRSHASKIMNVFSCAIDGLDRENGLQEVAKIVAHVGEYHA

DRKITKRSMVEMREVLTEKLIEVCHLDDEAIQAWSNLLDIVWCIMFDC

Nucleotide sequences were isolated in a manner similar to the amino acid results, with the exception that instead of regular expressions, custom code was written to find the correct region from all possible combinations of start-stop CDS within the sequence. Nucleotide sequences were verified as matching the amino acid sequence by "back-translating" the nucleotide sequence and comparing. If necessary, "extra" nucleotides at the end of sequences were trimmed to enforce codon boundaries - CDS lengths should always be a multiple of 3. Finally, the nucleotide sequence was written to a FASTA file in a format similar to the amino acid FASTA file. As above, with *Procladius villosimanius*:

>Procladius villosimanus:GGAY01021822.1

ATGAAAACGCTGACGCCGGAACAAGTGGCGATCATTAAGCGTACATGGGAACCAATTCAGTCGGATCACTTCAATAAGGGAGAGAAGATTTTGTTTGCTTATTTCGAGAAATATCCGATTAATCAGGAAAAGTTTCCAGCTTTTAGAAATACACCTCTTTTATTGTTGAAAGGAACTCCAGGTTTTAGATCTCACGCTTCGAAAATTATGAACGTTTTTTCCTGTGCAATCGATGGTTTGGATCGCGAAAATGGATTACAGGAAGTAGCAAAGATTGTCGCACATGTAGGAGAGTATCACGCTGACCGTAAAATCACCAAAAGATCAATGGTTGAAATGCGTGAAGTTTTAACGGAAAAACTCATAGAAGTGTGTCATTTGGATGATGAGGCGATACAAGCTTGGAGTAATCTTTTAGACATCGTGTGGTGCATCATGTTCGATTGTTTCGAT

>Procladius villosimanus:GGAY01021821.1

ATGGGAAATGTCATAACAAATATTCAAAAGAAAATCCATCCATCTGAAAATATTCCGATGAAAACGCTGACGCCGGAACAAGTGGCGATCATTAAGCGTACATGGGAACCAATTCAGTCGGATCACTTCAATAAGGGAGAGAAGATTTTGTTCGCTTATTTCGAGAAATATCCGATTAATCAGGAAAAGTTTCCAGCTTTTAGAAATACACCTCTTTTATTGTTGAAAGGAACTCCAGGTTTTAGATCTCACGCTTCAAAAATTATGAACGTTTTTTCCTGTGCAATTGATGGTTTGGATCGCGAAAATGGATTACAGGAAGTAGCAAAGATCGTCGCACATGTAGGAGAGTATCACGCTGACCGTAAAATCACCAAAAGATCAATGGTTGAAATGCGTGAAGTTTTAACGGAAAAACTCATAGAAGTGTGTCATTTGGATGATGAGGCGATACAAGCTTGGAGTAATCTCTTAGACATCGTGTGGTGCATCATGTTCGATTGT

# References

1. Python Software Foundation. Python Language Reference, Versions 3.6 and 3.7. 2019.

2. Cock PJA, Antao T, Chang JT, Chapman BA, Cox CJ, Dalke A, et al. Biopython: Freely available Python tools for computational molecular biology and bioinformatics. Bioinformatics. 2009;25: 1422–1423. doi:10.1093/bioinformatics/btp163

3. R Core Team. R: A language and environment for statistical computing, Version 3.6.1. Vienna, Austria; 2013. Available: http://www.r-project.org

4. R Studio Team. RStudio: Integrated Development for R. Boston, MA: RStudio, Inc.; 2015. Available: http://www.rstudio.com

5. National Center for Biotechnology Information [Internet]. NCBI Transcriptome Shotgun Assembly Database. 2018 [cited 1 Jun 2018]. Available: https://www.ncbi.nlm.nih.gov/Traces/wgs/?term=tsa

6. Xue Z. Convert NCBI taxonomy dump into lineages. In: Github [Internet]. 2019 [cited 1 Jun 2018]. Available: https://github.com/zyxue/ncbitax2lin

7. Thurmond J, Goodman JL, Strelets VB, Attrill H, Gramates LS, Marygold SJ, et al. FlyBase 2.0: The next generation. Nucleic Acids Res. 2019;47: D759–D765. doi:10.1093/nar/gky1003

8. National Center for Biotechnology Information [Internet]. Non-redundant (nr) database. 2019 [cited 1 Jun 2018]. Available: ftp://ftp.ncbi.nlm.nih.gov/blast/db/
