# Supplementary material for: Transcriptomes reveal expression of hemoglobins throughout insects and other Hexapoda
Source: PLoS One. 2020 Jun 5;15(6):e0234272. doi: 10.1371/journal.pone.0234272 (PMC7274415; doi:10.1371/journal.pone.0234272)
Supplement: S1 Fig — Cladogram of all globin transcripts, including 18 chordate globins, from amino acid analysis in RAxML. Bootstrap values labeled at nodes. Colored tags near various taxa in tree indicate locations of previously known hemoglobin sequences included to assist in homology assessment (S2 Table)[‘globin’, red]. Hemoglobin types labeled as belonging to globin X and X-like (according to Blank and Burmester [52]) or insectahemoglobin lineages (i.e., corresponding to D. melanogaster globins 1, 2, and 3). (PDF) [file pone.0234272.s001.pdf]

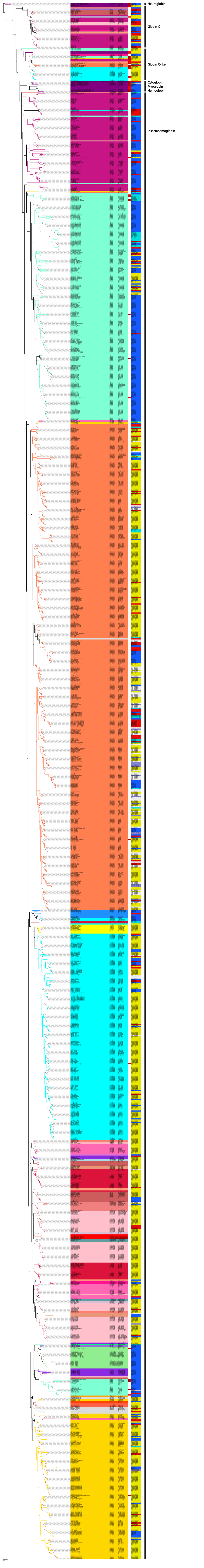

Neuroglobin
Globin X
Globin X-like
Cytoglobin
Myoglobin
Hemoglobin
Insectahemoglobin
